# Bright Light Therapy in Older Adults with Moderate to Very Severe Dementia: Immediate Effects on Behavior, Mood, and Physiological Parameters

**DOI:** 10.3390/healthcare9081065

**Published:** 2021-08-19

**Authors:** Nuria Cibeira, Ana Maseda, Laura Lorenzo-López, Isabel González-Abraldes, Rocío López-López, José L. Rodríguez-Villamil, José C. Millán-Calenti

**Affiliations:** Universidade da Coruña, Gerontology and Geriatrics Research Group, Instituto de Investigación Biomédica de A Coruña (INIBIC), Complexo Hospitalario Universitario de A Coruña (CHUAC), SERGAS, 15071 A Coruña, Spain; nuria.cibeira@udc.es (N.C.); laura.lorenzo.lopez@udc.es (L.L.-L.); i.gonzalez-abraldes@udc.es (I.G.-A.); rocio.lopez.lopez@udc.es (R.L.-L.); jose.luis.rodriguez-villamil@udc.es (J.L.R.-V.)

**Keywords:** bright light therapy, dementia, mood, behavior, oxygen saturation, heart rate

## Abstract

Bright light therapy (BLT) has demonstrated positive short- and long-term effects in people with cognitive impairment or dementia; however, the immediate impact of BLT sessions has been scarcely investigated. In this study, we aimed to explore the immediate effects of BLT on behavior, mood, and physiological parameters (oxygen saturation/heart rate) in a sample of institutionalized older adults with moderate to very severe dementia, with a median age of 85.0 (interquartile range, IQR, 82.0–90.0), being higher in men (87.0 years, IQR 80.0–94.0) than in women (84.5 years, IQR 82.0–89.5). The BLT protocol consisted of 30-min morning sessions of 10,000 lux, Monday through Friday, for 4 weeks. The physiological parameters were recorded immediately before and after each session by pulse oximetry. Mood and behavior were assessed before, after, and during the sessions using the Interact scale. Post-session Interact scores showed a significant decrease in the items *Tearful/sad* and *Talked spontaneously*, and a significant increase in the items *Enjoying self, active or alert*, and *Relaxed, content or sleeping appropriately*. Interact scores during the sessions reflected a significant decrease in the speech-related items. Both physiological parameters changed positively from before to after sessions. Our results suggest that BLT provides immediate positive effects on mood, stimulation level, and physiological parameters, as well as a trend toward decreased speech. More robust research is needed to further explore the immediate impact of BLT. This study is registered with Clinicaltrials.gov (NCT04949984).

## 1. Introduction

Dementia is one of the main causes of disability and dependence among older adults worldwide, constituting a public health priority due to its significant human and financial costs to society [1]. Oxidative stress, mitochondrial damage, and synaptic damage are all implicated in dementia pathogenesis, playing an important role in the cognitive impairment and memory loss of older individuals with Alzheimer’s disease (AD) [2,3,4]. Neurotransmitters are essential to maintain synaptic and cognitive function, and are also involved in altering the mood, depression, or anxiety of AD patients [5,6]. Aside from cognitive decline, dementia progression leads to the appearance of at least one neuropsychiatric symptom (NPS) in most individuals at some point during the course of their disease [7,8]. NPS, also known as behavioral and psychological symptoms of dementia (BPSD) [9], are defined as a series of signs and symptoms of disturbed perception, thought content, mood, and behavior that frequently occur in dementia and constitute part of the expression of the disease [10]. These symptoms mainly comprise delusions, hallucinations, agitation, depression, anxiety, apathy, irritability, euphoria, disinhibition, aberrant motor behavior, sleep and nighttime behavior disturbances, and changes in appetite and eating behaviors [11,12].

Treatment development for BPSD includes both pharmacological and nonpharmacological therapies. In clinical settings, pharmacological agents are frequently used in the management of these symptoms but show modest efficacy and significant serious side-effects [13,14], including increased risks of hospitalization, falls, and mortality [15]. Numerous guidelines and expert recommendations favor nonpharmacological interventions for the treatment of BPSD due to their significant impact on global BPSD outcome measurements and the lack of adverse events [16]. In fact, the American Geriatrics Society Beers Criteria Update Expert Panel recommends the use of nonpharmacological approaches as the first course of action, unless they have previously failed, are not feasible, or there is a substantial risk of harm to self or others [17]. Therefore, nonpharmacological therapies should be considered to be the first choice of treatment. Nonpharmacological interventions include cognitive stimulation, reminiscence therapy, reality orientation, validation therapy, animal-assisted therapy, exercise or physical activity, Snoezelen/multisensory stimulation, aromatherapy, music therapy, and light therapy, among others [18,19]. It is necessary for further research to continue to strengthen the evidence of their effectiveness [16].

In this study, we focus on bright light therapy (BLT). This therapy consists of the controlled application of certain levels of light that can be administered in different ways, including outdoor sunlight, light boxes, light visors worn on the head, ceiling lights, or dawn-dusk simulation [20]. BLT is reported to have positive effects in the management of BPSD, sleep disturbances, and circadian rhythms (see recent reviews [21,22]). Although numerous studies address the short- and long-term effects of this therapy in people with cognitive impairment or dementia, there is little research on its immediate effects. Furthermore, other sensory therapies—such as multisensory stimulation in a Snoezelen room or music therapy—are shown to provide immediate positive effects on measures of behavior and mood disturbances, as well as on certain physiological parameters such as heart rate or blood oxygen saturation [23,24,25]. Thus, it could be expected that BLT, as a sensory therapy, would also lead to improvements in mood, behavior, and physiological parameters. Therefore, the main objective pursued in this study was to explore the immediate effects of bright light therapy on behavior, mood, and physiological parameters (oxygen saturation and heart rate) in a sample of institutionalized older people with moderate to very severe dementia.

## 2. Materials and Methods

### 2.1. Design

This study was designed as a randomized controlled trial. Participants were stratified according to their cognitive status and were subsequently randomly assigned to the experimental group (BLT) or control group. In the analysis and exploitation of results, we only considered the response of the BLT group with regard to the stated objective since the control group did not undergo the intervention needed to complete Interact assessment during the study.

### 2.2. Participants

Participants were recruited from among the residents of a gerontological complex specializing in dementia (78.7% with cognitive impairment) and located in A Coruña, Spain. The gerontological complex has 70 older people in a day care-setting and 64 institutionalized older people in a nursing home. We selected all institutionalized participants (*n* = 64, with a mean age of 88.4 ± 8.0 years and a median age of 89.0 (interquartile range, IQR, 84.0–94.0)); 29.7% were men, with a mean age of 88.5 ± 9.4 years and a median age of 91.0 (interquartile range, IQR, 84.5–94.0), and 70.3% were women, with a mean age of 88.4 ± 7.5 years and a median age of 88.0 (interquartile range, IQR, 84.0–94.0). To delimit the sample, before the recruitment process, inclusion and exclusion criteria were established according to the existing literature. As inclusion criteria, participants were required to satisfy the conditions of being 65 years or older, diagnosed with dementia, scoring ≥4 points on the global deterioration scale (GDS) [26], and having signed the informed consent (directly or through their legal guardians). Based on a recent systematic review on the ocular safety of light therapy [27], individuals with increased ocular sensitivity to light (photosensitivity) or those with preexisting ocular abnormalities were excluded from the study. Residents who had any severe eye disorder that did not allow them to open their eyes, or involved very low visual acuity, were also excluded since light needed to enter the participant’s eyes to achieve the desired effects [28].

### 2.3. Procedure

The research protocol (code 2017/408) received favorable authorization from the Galician Research Ethics Committee of Xunta de Galicia, Spain, and was developed in accordance with the Declaration of Helsinki. The participants and their legal guardians were informed about the study and signed the corresponding informed consent. All the information transmitted was adapted to the level of comprehension of the participants to facilitate their understanding and comfort throughout the study.

The study was retrospectively registered with ClinicalTrials.gov (NCT04949984) on 2 July 2021. The study protocol was based on the implementation guidelines that we described in a recent review [22]. The BLT sessions were carried out in a quiet room of the gerontological complex especially enabled for their proper implementation and were conducted by two research psychologists specialized in gerontology. The devices used for the intervention were bright white light lamps providing an intensity of 10,000 lux. Four users participated in each session, with two users per lamp, seated in a comfortable chair with armrests which was 70 cm from the lamp. The sessions were 30 min/day, between 10:30 a.m. and noon, 5 days a week (Monday to Friday), for 4 weeks (total 20 sessions). During the sessions, while exposed to light, participants watched documentaries on neutral topics. Each session lasted 30 min, unless the participant expressed the desire to leave the room and could not be convinced to remain without generating agitation. Participants who responded negatively to light exposure were immediately withdrawn from the intervention. Only those sessions in which the participant remained for at least 80% of the expected time (≥22.5 min) were considered as sessions performed.

In every session, the researchers supervised the participants to ensure full compliance with the treatment and evaluated the physiological parameters, mood, and behavior of the participants. Once in the room, the investigators measured the heart rate (beats per minute) and blood oxygen saturation (SpO2) of each participant using a mobile finger pulse oximeter before and after the session.

Mood and behavior were assessed during the sessions and in the 10 min periods immediately before and after each session using the Interact scale [29] and its shortened version, named Interact short. The Interact scale, hereinafter referred to as Interact during, is made up of 22 Likert-scale items that score the frequency of occurrence (ranging from 1 (Not at all) to 5 (Nearly all the time)) of each type of mood and behavior during the sessions. Additionally, as qualitative information, any comments made by the participants about their session were recorded, as well as the sensation that the researchers believe the participants experienced during the session (relaxing, stimulating, leisure, improving communication, arousing curiosity, or unpleasant). The Interact short includes 12 Likert-scale items, with the same range as in the Interact during, recording the occurrence of each type of mood and behavior of the participants in the 10 min periods immediately preceding and following the session to measure any observable change.

### 2.4. Statistical Analysis

All data were analyzed using the SPSS statistical software package (version 26.0) (IBM, Armonk, NY, USA) and statistical significance was set at *p* < 0.05. The normal distribution of the variables was tested using the Shapiro–Wilk test. For subsequent analyses, we applied parametric or nonparametric tests depending on whether the assumption of normality was met or not for each measured variable. The Wilcoxon signed-rank test or paired *t*-test were employed to measure significant changes between the Interact short scores before and after the intervention sessions for each of the assessed mood and behavior items. Likewise, these same tests were used to analyze the evolution of the Interact during scores by comparing the average scores of the first week with those of the fourth week. In addition, the evolution of mood and behavior scores throughout the four weeks of treatment was analyzed with the Friedman test for related samples. The paired *t*-test was also used to determine the existence of any differences between before and after the sessions in the physiological parameters investigated. In the variables in which statistically significant differences were found, Cohen’s d or r values were used to report the effect size (ES) of these changes. The interpretation of the importance of the ES was made according to the benchmarks defined by Cohen [30] as follows: small ES (d = 0.2 or r = 0.1), medium ES (d = 0.5 or r = 0.3), and large ES (d = 0.8 or r = 0.5).

## 3. Results

### 3.1. Sample Characteristics

After applying inclusion and exclusion criteria, the total sample was reduced from 64 to 39 institutionalized older people (20 participants assigned to the control group and 19 to the BLT group). Table 1 shows their sociodemographic characteristics and their level of cognitive decline based on the scores obtained in the GDS. The categorical variables (gender, marital status, educational level, level of cognitive impairment) are expressed in terms of frequency and percentage, and age, as a continuous variable, is presented as the mean plus standard deviation (SD), along with the range of minimum and maximum values. The mean age of the participants was 86.3 ± 6 years, ranging from 75 to 98, with a median age of 85.0 (interquartile range, IQR, 82.0–90.0), being higher in men (87.0 years, IQR 80.0–94.0) than in women (84.5 years, IQR 82.0–89.5). The group was composed mostly of women (73.7%), and most participants were widowed (68.4%). Concerning educational level, few of the participants had higher education since most of them had received formal education for eight or fewer years (73.7%). Regarding the cognitive impairment measured by GDS, 31.6% of the sample presented moderate decline, 26.3% presented moderate to severe decline, another 26.3% presented severe decline, and finally, 15.8% of the participants presented very severe decline.

### 3.2. Mood and Behavior

Significant effects were detected between before and after the intervention sessions in 4 of the 12 items evaluated in the Interact short (see Table 2). The results of the Wilcoxon signed-rank test showed a significant reduction in the prevalence of the *Tearful/sad* item (*p* = 0.044) with a medium effect size (r = −0.32). The results of the paired *t*-test showed significant changes in the score of the *Talked spontaneously* item (*p* = 0.035) with a medium effect size (d = 0.52). Moreover, two items of the *Stimulation level* construct showed a significant increase in scores: *Enjoying self, active or alert* (*p* = 0.034) and *Relaxed, content or sleeping appropriately* (*p* = 0.001), with medium (d = −0.52) and large (d = −0.94) effect sizes, respectively.

To analyze the significance of the changes in the evolution of behavioral and psychological symptoms during the sessions, a comparison was made between the mean/median scores of the Interact during items of the four weeks of intervention. As can be seen in Table 3, scores reflected a significant decrease in the speech-related items, with large effect sizes. Scores were significantly lower for the *Comments or questions about activities/objects* item of the *Relating to the environment* construct. The results did not differ when analyzing the evolution of the four weeks versus the evolution between only the first and last week, with the same variables being significant in both comparisons.

Regarding the type of experience felt by the participants during the sessions, the evaluators recorded most of the sessions as leisure (32.98%), followed by relaxing (34.23%), stimulating (23.35), arousing curiosity (16.18%), and improving communication (6.43%). The researchers only perceived 6.32% of the sessions as unpleasant for the participants.

### 3.3. Physiological Parameters

**Figure 1:**
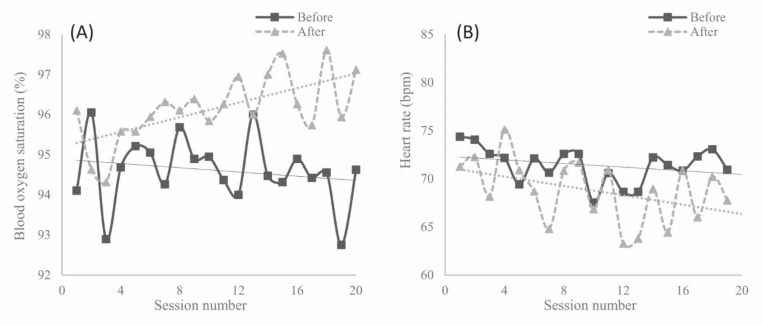
Records of biomedical parameters before and after light therapy intervention from session 0 (baseline) to 20 (post-trial): (**A**) blood hemoglobin oxygen saturation (%); (**B**) heart rate—beats per minute (bpm).

There were significant changes between before and after the sessions in both physiological parameters (see Figure 1). Participants demonstrated an increase in mean SpO2 values (*p* < 0.001, Figure 1A) and a decrease in mean heart rate (*p* < 0.001, Figure 1B) at the end of the sessions, with a large effect size of both changes measured (d = −1.33 and d = 1.10, respectively).

## 4. Discussion

The purpose of this study was to examine the immediate effects of a four-week BLT intervention program on the behavior, mood, oxygen saturation, and heart rate of institutionalized older patients with moderate to very severe dementia. The main results revealed promising evidence of the benefits of BLT on specific aspects of mood and behavior in participants both during and after the sessions, as well as positive effects on the physiological parameters analyzed.

As previously stated, mood and behavior were assessed using the Interact scale [29]. Although this scale was initially developed for the assessment of mood and behavior of patients with dementia during multisensory stimulation sessions in a Snoezelen room, its use has been extended to other nonpharmacological interventions such as music [24], art [31], and reminiscence therapies [32,33]. On this basis, the Interact scale was chosen for this study since its structure and form of application are also appropriate and compatible with the procedure of light therapy sessions.

The Interact short scores obtained by the participants before and after BLT sessions revealed, on the one hand, a significant decrease in the items of *Tearful/sad* and *Talked spontaneously*, and, on the other hand, a significant change in their stimulation level—specifically in the items *Enjoying self, active or alert*, and *Relaxed, content or sleeping appropriately*. Therefore, participants were less sad and less spontaneously talkative immediately after BLT sessions, which in turn could be related to the fact that they were also more relaxed, content, or sleeping appropriately. Concurring with some of these findings, other authors [34] investigating the immediate effects of BLT versus dim red light sessions found an immediate decrease in sleepiness and a tendency for mood improvement upon exposure to bright light. Other studies using 30-min BLT sessions found short-term positive effects on mood and agitation in people with dementia [35,36,37], with greater benefits observed in patients with severe dementia than in those with mild/moderate dementia [36].

The Interact during showed a significant decrease in the score of the *Making comments or asking questions about the activity* item, which makes sense due to the habituation of the participants to the intervention program as it progressed. Scores on the Interact during also reflected that as the weeks of intervention progressed, the participants talked less clearly, sensibly, and spontaneously and with shorter sentences, i.e., an overall decrease in speech during the sessions. These results may also be linked to the lower post-session scores on the *Talked spontaneously* item of the Interact short. None of the existing studies on BLT in the literature address the immediate effects that occur within sessions, so we cannot discuss our results from the perspective of previous studies.

Regarding physiological parameters, the measurements showed positive changes between before and after the sessions in the two analyzed parameters, with a significant increase in mean SpO2 and a significant reduction in mean heart rate. These changes may be related to the fact that participants were more relaxed, as shown by the Interact scores, since these biological responses are considered physiological indicators of a relaxation response [38,39]. Consistent with our findings, Choi et al. [40], who studied the immediate influence of different dim-colored lights—white, red, and blue—in healthy adults, also found a decrease in heart rate after exposure to the illumination. In the existing literature, another physiological parameter that has been studied with respect to light therapy is heart rate variability (HRV). A group of authors [41,42] found immediate positive effects of evening BLT on HRV in a sample of healthy young women. Similar findings were reported by Rechlin et al. [43] in patients diagnosed with major depression. Our results differ from another previous study in which no immediate effects of morning BLT on any of the physiological parameters analyzed were found [34]. Following the initial hypothesis of this study, our findings on the effects of BLT on the physiological parameters analyzed coincide with the positive effects of other sensory interventions reported in the existing literature, such as music therapy [24,25,44,45] and multisensory stimulation [23,24].

To the best of our knowledge, the immediate impact of BLT sessions on mood and behavior has been virtually unexamined previously, so the present study provides novel and relevant data for clinical practice. However, the results of this work should be regarded as preliminary and interpreted with caution due to certain existing limitations. This study is one part of a larger and more comprehensive BLT investigation. The current study only analyzed the immediate effects during and after BLT sessions, and the long-term effects should be further established. Therefore, these limitations stem mainly from the small sample size and the use of a single-arm intervention with no control group, both of which limit the generalizability of the results found. Additionally, mood and behavior were measured based on the observations of unblinded researchers, which may have resulted in researcher bias. Future research should address these limitations by employing a double-blind design in controlled studies with larger sample sizes. Another physiological parameter (determination of blood pressure at least three times during the sessions) could also be used to further assess the impact of bright light therapy.

## 5. Conclusions

The results of the present study suggest that a bright light therapy intervention program of 30-min sessions provides promising outcomes and immediate positive effects on mood, stimulation level, blood oxygen saturation, and heart rate. On the other hand, there was a tendency for a decrease in speech both during and after the intervention sessions. Further research is needed to strengthen these findings and to explore in depth the possible immediate effects of BLT.

## Figures and Tables

**Table 1:** Sociodemographic characteristics of the study population.

Characteristics of the Participants—*n* (%)		Total Sample (*n* = 19)
Gender	Female Male	14 (73.7) 5 (26.3)
Age	Mean age ± SD Age range	86.3 ± 6.0 75–98
Marital status	Single Married Widower Others	2 (10.5) 3 (15.8) 13 (68.4) 1 (5.3)
Educational level	Years of education ≤ 8 Years of education 9–17 Years of education > 17	14 (73.7) 2 (10.5) 3 (15.8)
Cognitive impairment—GDS ^1^	Stage 4. Moderate decline Stage 5. Moderate-severe decline Stage 6. Severe decline Stage 7. Very severe decline	6 (31.6) 5 (26.3) 5 (26.3) 3 (15.8)

^1^ GDS: global deterioration scale.

**Table 2:** Means (SDs) of Interact short scores before and after light therapy sessions.

Construct	Item	Before	After	*p*-value	ES
Mood	Tearful/sad ^a^	1.29 (0.46)	1.23 (0.43)	0.044 *	r = −0.32
	Happy/content ^b^	2.42 (0.88)	2.57 (0.97)	0.052	
	Fearful/anxious ^a^	1.20 (0.38)	1.08 (0.17)	0.069	
	Confused ^a^	1.21 (0.38)	1.17 (0.38)	0.128	
Speech	Talked spontaneously ^b^	2.67 (0.79)	2.53 (0.85)	0.035 *	d = 0.52
Relating to others	Related well to other staff/patients ^a^	3.86 (0.62)	3.84 (0.69)	0.717	
Relating to environment	Attentive/responsive/focused on environment ^a^	4.01 (0.61)	4.09 (0.56)	0.305	
Need for prompting	Did things from own initiative ^b^	1.88 (0.46)	1.86 (0.44)	0.707	
Stimulation level	Wandering, restless or aggressive ^a^	1.10 (0.18)	1.04 (0.07)	0.185	
	Enjoying self, active or alert ^b^	3.70 (0.64)	3.87 (0.60)	0.034 *	d = −0.52
	Bored, inactive or sleeping inappropriately ^a^	1.62 (0.66)	1.49 (0.64)	0.052	
	Relaxed, content or sleeping appropriately ^b^	3.57 (0.55)	3.87 (0.48)	0.001 **	d = −0.94

^a^ Wilcoxon signed-rank test; ^b^ paired *t*-test; * significant (*p*-value < 0.05); ** significant (*p*-value < 0.01); ES: effect size (Cohen’s d or r values).

**Table 3:** Means (SDs) of Interact during session scores for each week.

Construct	Item	Week 1	Week 2	Week 3	Week 4	*p*-Value	ES
Mood	Tearful/sad	1.40 (0.16)	1.27 (0.15)	1.16 (0.08)	1.12 (0.08)	0.675	
	Happy/content	2.41 (0.25)	2.11 (0.20)	2.15 (0.19)	2.05 (0.22)	0.425	
	Fearful/anxious	1.16 (0.05)	1.19 (0.15)	1.03 (0.07)	1.08 (0.09)	0.222	
	Confused	1.06 (0.04)	1.20 (0.08)	1.11 (0.08)	1.03 (0.05)	0.194	
Speech	Talked spontaneously	2.42 (0.13)	2.12 (0.29)	2.11 (0.24)	1.96 (0.27)	0.035 *	r = −0.43
	Recalled Memories	1.04 (0.07)	1.13 (0.12)	1.07 (0.11)	1.05 (0.12)	0.828	
	Spoke clearly	2.39 (0.14)	2.00 (0.37)	1.85 (0.22)	1.76 (0.06)	<0.001 ***	d = 0.95
	Spoke sensibly	2.39 (0.23)	1.94 (0.34)	1.84 (0.22)	1.76 (0.06)	0.001 **	d = 0.93
	Normal length sentences	2.37 (0.13)	1.95 (0.32)	1.85 (0.22)	1.76 (0.06)	<0.001 ***	d = 0.87
Relating to person	Held eye contact appropriately	3.36 (0.18)	3.35 (0.39)	3.22 (0.22)	3.16 (0.25)	0.729	
	Touching	3.03 (0.20)	3.17 (0.29)	3.23 (0.18)	3.04 (0.31)	0.923	
	Relating well	3.28 (0.24)	3.47 (0.41)	3.55 (0.15)	3.34 (0.20)	0.820	
	Co-operated	3.46 (0.19)	3.66 (0.45)	3.74 (0.13)	3.57 (0.18)	0.987	
Relating to environment	Tracked Observable Stimuli	3.43 (0.22)	3.51 (0.40)	3.35 (0.21)	3.27 (0.31)	0.907	
	Touched objects/equipment appropriately	3.37 (0.20)	3.55 (0.25)	3.45 (0.14)	3.37 (0.23)	0.674	
	Attentive to/responsive/focused on activity/objects	3.65 (0.27)	3.68 (0.19)	3.32 (0.32)	3.46 (0.15)	0.220	
	Comments/questions about activities/objects	2.13 (0.14)	1.57 (0.16)	1.59 (0.28)	1.42 (0.16)	<0.001 ***	r = −0.52
Need for prompting	Did things from own initiative	1.99 (0.10)	2.05 (0.19)	1.83 (0.07)	1.97 (0.17)	0.752	
Stimulation level	Wandering, restless or aggressive	1.20 (0.11)	1.05 (0.04)	1.11 (0.06)	1.12 (0.09)	0.204	
	Enjoying self, active or alert	3.57 (0.23)	3.56 (0.28)	3.33 (0.35)	3.50 (0.10)	0.526	
	Bored, inactive or sleeping inappropriately	2.18 (0.18)	2.09 (0.28)	2.43 (0.24)	2.12 (0.28)	0.439	
	Relaxed, content or sleeping appropriately	3.54 (0.15)	3.57 (0.13)	3.33 (0.24)	3.61 (0.19)	0.478	

* Significant (*p*-value < 0.05); ** significant (*p*-value < 0.01); *** significant (*p*-value < 0.001); ES: effect size (Cohen’s d or r values).

## Data Availability

The data presented in this study are available on request from the corresponding author.
